# A Preliminary Study on *Oxya fuscovittata* (Marschall) as an Alternative Nutrient Supplement in the Diets of *Poecillia sphenops* (Valenciennes)

**DOI:** 10.1371/journal.pone.0111848

**Published:** 2014-11-10

**Authors:** Arijit Ganguly, Ranita Chakravorty, Angshuman Sarkar, Dipak K. Mandal, Parimalendu Haldar, Julieta Ramos-Elorduy, Jose Manuel Pino Moreno

**Affiliations:** 1 Department of Zoology, Visva-Bharati University, Santiniketan, West Bengal, India; 2 Department of Statistics, Visva-Bharati University, Santiniketan, West Bengal, India; 3 Departamento de Zoología, Universidad Nacional Autonoma de Mexico, Mexico City, Distrito Federal, México; Universiti Sains Malaysia, Malaysia

## Abstract

Growth of the ornamental fish industry is being hindered by the scarcity of low cost feed; hence alternative protein supplements should be explored. In this context the present study aims to evaluate whether the grasshopper *Oxya fuscovittata* could be used as a supplement for fish meal in the diets of *Poecillia sphenops*, which is one of the most common ornamental fishes worldwide. The present work is divided into three phases: In the first phase proximate composition of the grasshopper is obtained and five diets are prepared where fish meal is gradually replaced by *Oxya* meal and named as control, D1, D2, D3 and D4. All the diets are formulated on iso-nitrogenous basis where the protein percentage is fixed at 400 g/kg. The second phase deals with feeding trial and in the third phase all the data of the feeding trial are subjected to a linear model. The feeding trial shows that the control, D1 and D2 fed fishes have almost similar results. The linear model proves that the variation in the indices are mainly due to replacement of fish meal by *Oxya* meal, not due to the variations of rice husk and mustard oil cake that are also used to formulate the diets of the present study. From the results two *Oxya* supplemented diets, i.e. D1 and D2 are proved to be almost equivalent to the control diet. Hence it is concluded that *Oxya* meal is able to replace 25% to 50% of fish meal from the diets of *P. sphenops*.

## Introduction

Fish farmers in general are facing difficulty in raising their fishes because of an alarming increase in the cost of feed which represents 60–70% of the total production cost [1]; and this is also true for ornamental fish industry. In concert with the high rising production cost, the market price of the ornamental fishes is also gaining height [2]. Moreover the conventional protein sources for fish industry i.e. fish meal, soy meal and other grains are being competed for by the human population as well [3]. Recently, with a global population boom the increasing demand and uncertain availability of these traditional protein sources resulted in the quest of other protein rich alternatives for inclusion into the fish diets [4], [5]. In this context, insects being nutritionally rich could be a possible alternative [1]. Among the edible insects, acridids (i.e. short-horn grasshoppers of family Acrididae of the order Orthoptera) might have a great future to be established as a sustainable mini-livestock. Acridids are suitable insects for consumption because according to the report of Xiaoming et al. acridids have a high nutritional value and can be used to formulate good quality feed for livestock [6]. But for a constant supply to the feed developing companies there is a need to build acridid farms, for which a suitable species should be found out. In this context Anand et al. reports an estimation of probable annual biomass that could be produced in acridid farms [7]. According to their conclusions *Oxya fuscovittata* (Marschall) is most suitable because being tetra-voltine, they can complete four life cycles annually and could produce a huge biomass. Keeping this in mind the present study aims to utilize *O. fuscovittata* as a supplement of fish meal in the diets of *Poecilia sphenops* (Valenciennes).

## Materials and Methods

The present work is divided into three phases; in the first phase analysis of nutrient composition of the ingredients are carried out, which is followed by feed formulation and diet quality assessment; in the second phase the feeding trial is conducted with the selected ornamental fish. During diet formulation fish meal is gradually replaced by *Oxya* meal; but to formulate diets on iso-nitrogenous basis, two other ingredients are needed to vary. Consequently the amounts of those ingredients differ from diet to diet. In this context it is hard to infer that the significant differences in the results of feeding trial is due to replacement of fish meal by *Oxya* meal, or due to the variations in the amounts of the other major ingredients. Hence, in the third phase all the data of the feeding trial are fitted to a linear model, to conclude which ingredient has greater effect on the variations of the observed indices during the feeding trial.

The present work has been conducted according to the rules of “Institutional Animal Ethics Committee” of Visva-Bharati University, and approved by the committee according to the Indian law.

### Selection of model ornamental fish

To accomplish the present work we have chosen *P. sphenops* (more commonly “black molly”) which could be easily reared, could complete life cycle within a short period and easy to breed. Moreover, it is one of the most common ornamental fishes to be seen in the aquariums.

### Diet formulation

For the formulation of various supplementary diets *Oxya* meal, fish meal (dried *Harpadon nehereus* F. Hamilton 1822), mustard oil cake (MOC) and rice husk are used as major ingredients. Carboxymethyl cellulose (CMC) is used as a binder for the diets and calcium, table salt (NaCl) and vitamin-mineral mixture are used as additive. First of all proximate composition of the major ingredients are carried out according to AOAC methods [8]. Nitrogen is estimated using Kjeldahl method and crude protein is calculated by multiplying the amount of nitrogen with the factor 6.25 (988.05). Crude fat is estimated with soxhlet apparatus (920.39). Ash contents are determined by subjecting the samples to muffle furnace at 550°C for 6 hrs (942.05). The amount of carbohydrate content is calculated by difference method (100– sum of protein, fat and ash).

Five diets are prepared gradually replacing fish meal by *Oxya* meal with the ratio of 100∶0, 75∶25, 50∶50, 25∶75 and 0∶100. The diet having “fish meal: *Oxya* meal” with the ratio of 100∶0 is considered as control because there is no supplement of *Oxya* meal. The other diets are named D1, D2, D3 and D4 respectively. The amount of ingredients of all of the formulated diets are mixed in such a way so that each of them contain about 400 g/kg crude protein, because according to literature mollies need this much protein in their diets for optimal growth [9]. Maintaining the amount of protein by adjusting the proportion of ingredients is done by the Pearson’s square method. After formulation, diets are first made to dough forms that are subjected to a pelletizer (RJK-FFF-PM-52, Rajkumar Agro Engineers Pvt. Ltd, India), to make floating type pellets.

### Assessment of diet quality

Crude protein, crude fat (ether extract), carbohydrate and total ash (TA) are estimated according to AOAC as already mentioned [8]. Energy contents of the diets are estimated by oxygen bomb calorimeter and expressed in kJ/g. Protein to energy (P/E) ratio is an important measure for the quality assessment of the formulated diets and it is expressed as “mg of protein/kJ of energy”. Feed acceptability test is assessed as the time taken by the first fish to strike the first particle after the diet dropped into the tanks [10]. The time is recorded in seconds using a stop watch.

### Water quality

Analysis of water of the experimental tanks has been done fortnightly in terms of temperature, pH, dissolved oxygen (DO) and hardness. Temperature and pH is measured using a combined pH meter and thermometer (ZPHI-9100), Zico India Ltd., but DO and hardness is measured by titration according to APHA [11].

### Collection of experimental species

Same variety of twenty females and ten males of *P. sphenops* are bought from the local market of Bhubandanga, Santiniketan, West Bengal, India. They are reared in the laboratory using a *Spirulina* based diet till the females give birth to sufficient amount of hatchlings. One hundred and fifty (150) fingerlings of similar initial weight (about 0.01 g) are selected to start the feeding experiment, and are kept for 7 days of acclimation period prior to feeding.

### Experimental setup

150 fingerlings are divided into 5 sets with 3 replications having 10 individuals in each group. They are placed in 15 experimental tanks of 26 L capacities. Half of the total volume of water is changed daily from each tank. The tanks are completely drained and thoroughly scrubbed once a week to prevent algal growth. Adult body weight and length are measured at the end of experiment after 176 days.

### Feeding the fishes

The experimental fishes are fed with known amount of oven dried feed twice daily at 6.30 and 18.30 hrs for five minutes. The uneaten diets are siphoned off and oven dried. The amount of dried feed consumed is calculated by subtracting the left over dried feed from the known amount of dried feed offered.

### Estimation of consumption, utilization, survival, growth and reproductive potential of *P. sphenops* when fed with the formulated diets

Growth is measured in terms of specific growth rate (SGR) and condition factor (K) according to De Silva & Anderson, and Moyle & Cech using the following equations [12], [13].
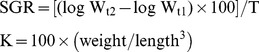



Food consumption and utilization is measured in terms of protein efficiency ratio (PER), feed conversion ratio (FCR), and feed conversion efficiency (FCE) as suggested by De Silva and Anderson using the following equations [12].
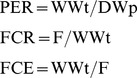
[Where W_t1_ = initial weight, W_t2_ = final weight, T = number of days, F = average dry weight of food ingested per individual, WWt = wet weight gained and DWp = dry weight of protein in feed].

Survival is measured in terms of percentage of individuals alive up to sexual maturity, whereas reproductive potential is measured in terms of total number of offspring born per female in the experimental sets.

### Statistical analysis phase 1 and 2

All the data are presented as means ± SD. Each variable is first subjected to one way analysis of variance (ANOVA). Thereafter separation of mean values according to significance is done within each variable by Tuky’s range test. All the statistical analyses are carried out using S Plus version 4.0 and PAST version 3.02.

### Statistical analysis phase 3

To make sure whether the responses obtained in the feeding trial are due to the replacement of fish meal by *Oxya* meal or due to the variation of rice husk and mustard oil cake, the data of the response indices are fitted to the following linear model using MATLAB program (MATLAB 7.1)–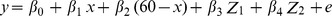
[Where, y = response indices, 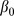  = effect of some constant factors that are identical for all the formulated diet fed sets, 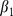  = effect due to x, x = *Oxya* meal, 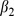  = effect due to (60-x), (60-x)  = fish meal, 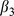  = effect due to 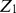, 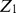  = rice husk, 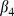  = effect due to 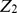, 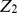  = mustard oil cake, e = error].



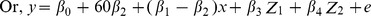



This equation could be simplified as–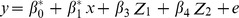
[Where, 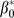  = (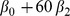), and 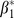  = (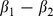). In other words according to this model, 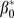, 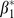, 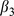, and 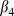 are the “estimate of the regression coefficients” of the effects due to their corresponding factors (i.e. intercept or the constant effect, *Oxya* meal that replaced fish meal, rice husk and mustard oil cake respectively) on the response indices].

In the next step, “estimate of the regression coefficients” are individually subjected to student t-test to know whether their corresponding factors have significant effect on the response indices. If the null hypothesis is accepted, one could conclude that the corresponding factor has no significant effect and if the null hypothesis is rejected, one could conclude that the corresponding factor has significant effect on the response.

After this test the four major factors of the formulated diets (i.e. *Oxya* meal, fish meal, rice husk and mustard oil cake) are divided into two groups–

replacement of fish meal by *Oxya* meal = Group1
rice husk + mustard oil cake = Group2

Then the effect of Group1 on the response indices are statistically compared with the same of Group2, to conclude which group has greater effect on the variation of the response indices. This is done by comparing the sum squares (SS) of the effect of Group1 and Group 2 because SS represents the sum of squared difference from the mean, and this difference gives rise to variance. This part is carried out using the same model already programmed in MATLAB.

## Results

### Diet formulation

Proximate compositions of the major ingredients i.e. rice husk, *Oxya* meal, MOC and fish meal are tabulated in table 1. It reveals that crude protein level is highest in *Oxya* meal (more than 640 g/kg) and crude fat is highest in fish meal (more than 90 g/kg). Contents of carbohydrate and total ash show higher values for rice husk. After getting the proximate composition of the major ingredients, their required proportions in the formulated diets are calculated. The final compositions of the diets are depicted in table 2.

**Table 1:** Proximate composition of main ingredients.

Ingredients	CP(g/Kg±SD)	EE(g/Kg±SD)	C(g/Kg±SD)	TA(g/Kg±SD)
Fish Meal	483.1±07.2c	92.8±19.0b	381.7±10.1b	42.4±17.5a
*Oxya* Meal	640.7±02.3d	64.8±03.1a	241.8±04.7a	52.7±0.9a
Rice Husk	54.42±01.5a	49.0±08.0a	742.3±11.8d	154.3±11.1b
Mustard Oil Cake	306.3±12.8b	52.7±12.5a	606.5±12.2c	34.5±11.9a

Note: CP = crude protein, EE = ether extract (crude fat), C = carbohydrate, TA = total ash. Values with different letters within a column are significantly different (P<0.001) using Tukey’s range test.

**Table 2:** Ingredient composition and quality of formulated diets.

Ingredients	Control	D1	D2	D3	D4
Fish meal	600.00 g	450.00 g	300.00 g	150.00 g	0.00 g
Acridid meal	0.00 g	150.0 g	300.0 g	450.0 g	600.0 g
Mustard oil cake	339.00 g	267.80 g	173.70 g	80.10 g	12.70 g
Rice husk	11.00 g	82.20 g	176.30 g	269.90 g	337.30 g
Carboxy methyl cellulose	30.00 g	30.00 g	30.00 g	30.00 g	30.00 g
Vitamin-Mineral mixture^a^	7.00 g	7.00 g	7.00 g	7.00 g	7.00 g
NaCl	8.00 g	8.00 g	8.00 g	8.00 g	8.00 g
Calcium additive	5.00 g	5.00 g	5.00 g	5.00 g	5.00 g
Total	1 Kg	1 Kg	1 Kg	1 Kg	1 Kg
Formulated diet quality					
CP (g/Kg ± SD)	399.4±6.2a	400.1±5.3a	401.1±6.7a	399.9±8.1a	400.6±7.6a
EE (g/Kg ± SD)	63.1±11.3a	57.3±9.8a	52.7±12.3a	61.2±11.8a	52.3±8.7a
C (g/Kg ± SD)	498.4±7.7a	517.1±4.8b	522.1±10.1b	523.1±8.6b	523.3±4.4b
TA (g/Kg ± SD)	44.1±4.6b	27.5±3.1a	25.2±4.8a	21.8±3.6a	23.8±2.7a
Energy (kJ/g ± SD)	18.94±0.06a	19.15±0.04b	19.15±0.07b	19.39±0.06c	19.19±0.05b
P/E(mg protein per kJ ± SD)	20.82±0.19a	20.89±0.13a	21.14±0.11b	20.61±0.08a	21.18±0.19b
Striking time (in seconds)	6.50±1.22a	6.72±0.68a	7.06±1.57a	7.24±0.28a	6.36±0.16a

Note: CP = crude protein, EE = ether extract (crude fat), C = carbohydrate, TA = total ash, P/E = protein to energy ratio. Values with different letters within a column are significantly different (P<0.001) using Tukey’s range test.

a Provided *per* Kg of diet: Vitamin A (as Acetate) 69000 IU, Vitamin D3 6900 IU, Vitamin E Acetate 172.5 mg, Ascorbic Acid 1035 mg, Thiamine Mononitrate 69 mg, Riboflavine 69 mg, Pyridoxine HCl 20.7 mg, Cyanocobalamine 103.5 mcg, Niacinamide 690 mg, Vitamin K 1.7 mg, Calcium Pentothenate 112.4 mg, Calcium Phosphate 890.1 mg, Magnesium Oxide 414 mg, Ferrous Fumerate 221.1 mg, Zinc (from zinc sulphate) 15.2 mg, Copper (from copper sulphate) 23.4 mg, Manganese (from manganese sulphate) 14 mg, Sodium Molybdate 1.7 mg, Sodium Borate 6.1 mg.

### Assessment of diet quality

Quality of the formulated diets is presented in table 2. Almost 400 g/kg of crude protein level is obtained in each of the diets with no significant difference (p>0.05, Tuky’s range test). Similarly the values of crude fat (ether extract) again show statistically insignificant variation. The values of carbohydrate contents are also very similar; however, in the control diet it is slightly lower than the others (p<0.05, Tuky’s range test). Total ash has a result which is reciprocal to the values of carbohydrate where the control diet shows statistically higher (p<0.001, Tuky’s range test) results. Energy is quite similar, but the control and D3 has significantly lowest and highest mean values respectively (p<0.001, Tuky’s range test). Protein to energy ratios (P/E) are almost the same (nearly 21 mg/kJ) for all the diets. Results of the acceptability test reveals that the time taken by the first fish to strike the first particle of the formulated diets is within about 6–7 seconds for each case. Hence no significant variation is obtained by Tuky’s range test.

### Assessment of water quality of the experimental tanks

The temperature, pH, dissolved oxygen and hardness of water during the feeding period are tabulated in table 3. Temperature of all the experimental tanks range from 21.4 to 30.5°C with a mean value of 27.18°C. The water is found to be slightly alkaline as the pH range falls between 7.34–7.71 with a mean value of 7.52. Dissolved oxygen has an average value of 3.61 mg/L and it ranges between 3.14–3.82 mg/L in all the experimental tanks. Assessment of hardness reveals that the water of the experimental tanks is hard with a mean value of 165.67 mg/L that ranges between 162.4–167.6 mg/L.

**Table 3:** Water quality of the experimental tanks.

Parameters	Range	Mean ± SD
Temperature (°C)	21.40–30.50	27.18±1.25
pH	7.34–7.71	7.52±0.48
Dissolve Oxygen (mg/L)	3.14–3.82	3.61±0.52
Hardness (mg/L)	162.40–167.60	165.67±2.20

### Consumption, utilization, survival, growth and reproductive potential of *P. sphenops* when fed with the formulated diets

The results of survival, SGR, PER, FCR, FCE, K and reproductive potential of *P. sphenops* are summarized in table 4. More than 80% survival is obtained for all the diet fed sets and they do not vary significantly. But for the other indices as a whole there is a tendency of the control diet, along with D1 and D2 fed *P. sphenops* to have similar results that are significantly better than D3 and D4 fed groups. In case of specific growth rate (%SGR) the values vary insignificantly (p>0.05, Tuky’s range test) between the control, D1 and D2 fed sets and these values are higher than D3 and D4 fed ones. The values of feed conversion ratio (FCR) also show better results for the control, D1 and D2 diets as the results are significantly lower (p<0.05, Tuky’s range test) than the groups fed with D3 and D4. On the contrary unlike SGR and FCR, the results of protein efficiency ratio (PER) and feed conversion efficiency (FCE) statistically vary between the control, D1 and D2 fed sets. For PER among these three diets D1 fed groups show highest values (p<0.05, Tuky’s range test) followed by control and D2 fed groups. For FCE, though the values are insignificant between control and D1 fed groups, the sets fed with D2 shows a slightly lower value (p<0.05, Tuky’s range test). Condition factor (K) also shows a similar trend, values vary insignificantly between control, D1 and D2 fed mollies and then it gradually decreases in D3 and D4 fed sets. Each female of all the experimental tanks are found to give birth to 13–16 offspring. On an average the values are insignificant (p>0.05, ANOVA, Tuky’s range test) when data are compared between the sets fed on different formulated diets.

**Table 4:** Results of Survival, SGR, PER, FCR, FCE, K and reproductive potential of *P. sphenops* fed with various formulated diets.

Response indices	Control	D1	D2	D3	D4
% Survival	84.19±2.36a	83.78±2.11a	83.93±1.82a	82.97±2.32a	83.31±1.97a
SGR	1.52±0.10c	1.52±0.08c	1.5±0.17c	1.26±0.06b	1.04±0.14a
PER	14.97±0.28d	15.97±0.24e	12.18±0.40c	3.16±0.26b	1.13±0.05a
FCR	1.16±0.03a	1.14±0.02a	1.18±0.05a	1.86±0.08b	2.21±0.11c
FCE	3.40±0.23d	3.62±0.14d	2.76±0.14c	0.97±0.13b	0.45±0.09a
K	1.66±0.0187c	1.55±0.192c	1.94±0.206c	1.01±0.097b	0.55±0.016a
Reproductive potential/female	14.33±1.53a	14.67±1.15a	15.67±0.58a	14.33±1.15a	15.33±0.58a

Note: SGR = Specific growth rate, PER = Protein efficiency ratio, FCR = Feed conversion ratio, FCE = Feed conversion efficiency, K = Condition factor. Values are means ± SD. Values with different letters within a row are significantly different (P<0.001) using Tukey’s range test.

### Linear model of response variables

The estimate of the regression coefficients of the effects of different factors used in the linear model on each response index are depicted in table 5. Survival and reproductive potential are excluded from this analysis as they vary insignificantly from diet to diet (p>0.05). Values of the student t-test that are conducted to know whether their corresponding factors have significant effect on the response indices are also presented in the same table. The insignificant p values (p>0.05, Tuky’s range test) show that the null hypothesis is rejected for all. This means all the factors in question (i.e. replacement of fish meal by *Oxya* meal, rice husk and mustard oil cake) has certain amount of effect on the variation of the response data that are obtained during the feeding trial. Now, as it has been evident that all the factors in question are playing some role on the variation of the response data, in the next step it is essential to find out which factor is playing greater role. For this purpose the sum of squares (SS) of the effects due to replacement of fish meal by *Oxya* meal (Group1) is compared with the sum of squares (SS) of the effects due to rice husk and mustard oil cake in combination (Group 2). Results of the sum of squares along with their corresponding p values have been tabulated as table 6. This table has revealed that only excluding PER, for rest of the cases, Group1 has much greater effect on the variations in response data that are obtained during the feeding trial. This proves that the effect of replacement of fish meal by *Oxya* meal has greater influence on the variation of response than the effect of rice husk and mustard oil cake in combination.

**Table 5:** Estimate of the regression coefficients of the effects of different factors used in the linear model on each response variable.

Response indices	Estimate of the regression coefficients			
	Intercept [P value]	Replacement [P value]	Rice husk [P value]	Mustard oil cake [P value]
SGR	0.0003 [0.4999]	0.0080 [0.4970]	0.0055 [0.4979]	0.0047 [0.4982]
PER	0.0004 [0.4998]	0.0074 [0.4972]	0.0107 [0.4960]	0.0044 [0.4983]
FCR	0.0002 [0.4999]	0.0089 [0.4967]	0.0016 [0.4994]	0.0051 [0.4981]
FCE	0.0004 [0.4999]	0.0070 [0.4974]	0.0090 [0.4966]	0.0042 [0.4984]
K	0.0003 [0.4999]	0.0077 [0.4971]	0.0067 [0.4975]	0.0045 [0.4983]

Note: SGR = Specific growth rate, PER = Protein efficiency ratio, FCR = Feed conversion ratio, FCE = Feed conversion efficiency, K = Condition factor, Intercept = constant effect, Replacement = *Oxya* meal that replaced fish meal, P value = Area under the t distribution above the observed t value of the student t-test that was conducted to know whether their corresponding factors had significant effect on the response.

**Table 6:** Comparison between the sum of squares of the effects due to Group1 and Group2 on the response indices.

Response Variables	Sum of squares due to Group1 [×10^3^]	Sum of squares due to Group2 [×10^3^]	P-value of the ratio of sum of squares
SGR	215.9000000	0.7788000	<0.001
PER	0.9156000	0.2053000	0.0883
FCR	543.9200000	26.7680000	<0.01
FCE	21.7300000	3.3322000	<0.01
K	135.9900000	3.4697000	<0.01

Note: SGR = Specific growth rate, PER = Protein efficiency ratio, FCR = Feed conversion ratio, FCE = Feed conversion efficiency, K = Condition factor, Group1 = replacement of fish meal by *Oxya* meal, Group2 = rice husk and mustard oil cake in combination.

## Discussion

It is already known that mollies prefer hard water with a pH range of 7.5–8.2, and 18°C−28°C is the suitable temperature for them [14]. In the present study the result of water quality reveals that the water in the experimental tanks is hard with a temperature and pH within the optimum range. However, dissolved oxygen is a little lower than the optimum need (i.e. above 5 mg/L) for most of the fishes [15], [16]. The main cause behind this lower DO may be because no aerator is used in the tanks. However, mollies have been reported to get adapted to hypoxic conditions as they could tolerate a level of DO as low as 1 mg/L [17]. In this context it could be stated that the water condition of the experimental tanks are ideal for rearing mollies.

Protein is one of the most important constituents in feed for fish growth. Before designing any experiment on the effect of formulated diets on growth of fishes, the dietary percentage of protein needed for their optimal growth should be obtained first [18]. Shim and Chua worked on poeciliids like *Poecilia reticulata* Peters 1859 and according to them 300–400 g/kg of protein in feed is optimum for their growth [19]. Parker also opines that 400 g/kg dietary protein is optimum for mollies [9]. Hence 400 g/kg of crude protein is fixed for all the formulated diets in the present study. According to some workers, 100–200 g/kg of lipid in fish diets gives optimum growth rates without producing an excessive fatty carcass [20]. In this context it may seem that the diets of the present study contain a little lower amount of fat (near about 50–60 g/kg). However, report of Fernando et al. states that poeciliids are fed with diets having 30–70 g/kg of fat in Singapore [21]. This report supports the view that the formulated diets of the present study contain sufficient amount of fat. It is well known that fish apparently do not have carbohydrate requirement, but it could be metabolized by many fishes and usually herbivorous fishes can metabolize carbohydrates better than carnivorous species [22], [23]. According to the report of National Research Council warm water fish (such as mollies) are able to use high carbohydrate levels as an energy source [24]. This phenomenon is advantageous because carbohydrate sources are inexpensive as seen in the present case where all of the formulated diets contain a high amount of carbohydrate. Protein to energy (P/E) ratio is another important measure that gives us a clear picture whether the balance between protein and energy contents is maintained in the diets in proper amount. According to some authors for achieving maximum growth, the P/E ratio in fish diet should range from 19–22 mg/kJ [25]–[27]. These reports are encouraging because in the present case all of the formulated diets have P/E value of nearly 21 mg/kJ.

Before feeding trial it is necessary to be sure whether the experimental fish recognizes the diets as a palatable one. The results of low feed striking time with insignificant variation positively indicate about the palatability of all the formulated diets. The feeding trial reveals that the results of growth, food consumption and utilization are very similar when fed on control, D1 and D2. On the contrary the values when fed on D3 and D4 are of a lower grade. Among the very little information in literature on using insects as alternative protein supplement for fish, satisfactory results for *Heterobranchus longifilis* Valenciennes 1840 has been reported, where 50% fish meal is replaced by termite meal [28]. Martinez et al. also report satisfactory results when 30% cockroach meal is added to formulate diets for Japanese carp [29]. Along with food consumption, utilization and growth, the measurement of condition factor (K) is also important as it could express the final health of the adult individuals in terms of length and weight at the end of the feeding trial. Results of the condition factor follow a similar trend as observed in case of the other indices already mentioned, because here also the results of control, D1 and D2 diets show ideal results (more than 1.5 [30], [31]). Unsatisfactory results are obtained in case of D3 and D4 fed groups might be because of mollies cannot tolerate a composition where more than 50% of fish meal is replaced by the *Oxya* meal. Or this could be an effect of variations in the amount of rice husk and mustard oil cake.

To clear the doubt whether the difference in response variables of feeding trial are due to replacement of fish meal by *Oxya* meal, or due to the effect of change in the amount of rice husk and mustard oil cake, a statistical explanation is important. In this regard results of the linear model quite clearly reveal that only excluding PER, variations of all the indices under consideration occur mainly due to the replacement of fish meal by *Oxya* meal. Here it should be kept in mind that PER is actually a measure of weight gain with respect to the amount of protein consumed through food. The statistical model shows that *Oxya* meal, fish meal, rice husk and mustard oil cake, all have certain effect on the response variables; it is quite clear that all these four ingredients contribute in the protein part of the formulated diets. In this context it could be explained that may be a significant amount of protein contribution by all the four major ingredients leads to an insignificant result when the effect of replacement of fish meal by *Oxya* meal is compared with the combined effect of rice husk and mustard oil cake in case of PER. Results of the present work is encouraging because it is quite clear from this study that at least up to 50% of fish meal could be effectively replaced by *Oxya* meal. This amount of replacement will have no negative effect on the growth and reproduction of *P. sphenops.* Moreover, it is also clear that the variations on the response indices are mainly due to this replacement and not due to the varied amount of rice husk and mustard oil cake.

The results of the present study as a whole support the view of rearing *O. fuscovittata* in “acridid farms” that will easily and constantly provide nutritionally rich animal protein supplement to the aqua-feed developers to formulate supplementary diets for ornamental fishes making the industry more viable. Moreover, if the concept of “acridids as alternative food source” is popularized it could lower the rate of overexploitation of fish meal. Consequently the “demand: supply” ratio of the fish meal could come down, resulting a lower market price of the fish meal also. However, similar studies with various proportions of *Oxya* supplement in the diets of other ornamental fishes are necessary in future. These works also should emphasize on obtaining the digestibility of the formulated diets, which is lacking in the present work. Then only one could be more assured of the suitability of *Oxya* meal as an effective alternative protein source.
